# Experiencing physical warmth affects implicit attitudes and altruistic behavior toward outgroup in females

**DOI:** 10.1186/s13104-017-2972-3

**Published:** 2017-11-29

**Authors:** Takeru Miyajima, Xianwei Meng

**Affiliations:** 1 Graduate School of Human-Environment Studies, Kyushu University, 6-19-1 Hakozaki, Higashi-ku, Fukuoka, 812-8581 Japan; 2 Japan Society for the Promotion of Science, Kojimachi Business Center Building, 5-3-1 Kojimachi, Chiyoda-ku, Tokyo, 102-0083 Japan

**Keywords:** Embodied cognition, Physical warmth, Interpersonal warmth, Intergroup bias, Gender difference, Implicit association test, Helping behavior

## Abstract

**Objective:**

Experiencing physical warmth has been demonstrated to influence interpersonal warmth. However, the effects of this metaphorical link in an intergroup context is not clear. The current study aimed to investigate the effect of physical warmth on implicit attitudes and behavior toward outgroup members in a Japanese–Chinese intergroup context. After touching either a warm or cold cup for 3 min, the Japanese participants were required to complete the single-target implicit association test, which aimed to measure their implicit attitudes toward imagined Chinese people, and to express their willingness to participate in the experiments of a Chinese individual whom they interacted directly without compensation, aiming to measure their prosocial behavior toward a real outgroup member.

**Results:**

The results demonstrated that female participants who touched the warm (vs. cold) cup showed more positive attitudes and helping behavior toward the Chinese individual. Furthermore, the correlation between those attitudes and helping behaviors supports the effects of enhanced implicit attitudes and further suggests that experiencing physical warmth could increase prosocial response to outgroup members in real interactions. However, the male participants showed a reversed, but not statistically significant, effect of physical warmth on the implicit attitude.

**Electronic supplementary material:**

The online version of this article (10.1186/s13104-017-2972-3) contains supplementary material, which is available to authorized users.

## Introduction

Research on embodied cognition has shown that perceived interpersonal warmth (e.g., attitude toward a person) can be moderated by the experience of physical warmth (e.g., touching a warm cup) [1]. Despite the possible common biological basis (e.g., the insular cortex) of and the experience-based association (from an “attachment theory” view) between physical and social warmth [2, 3], the underlying mechanisms of the metaphorical links remain unclear. Examinations on whether the metaphorical links extend beyond close or neutral others to outgroup members might provide further insights as evaluations of outgroup members are possibly related to the mentioned roots [4, 5] and the embodied reactions to ingroup and outgroup members’ behaviors (e.g., mimicry) vary greatly [6].

Breines [7] examined whether physical warmth can improve implicit attitudes toward outgroup members. European–American participants wrapped a warm or cold compress around their nondominant forearms and completed the implicit association tests (IATs). The tests assessed participants’ implicit attitudes toward African–Americans *relative to* European–Americans (dual-target IAT; Exp1) or implicit attitudes *directly toward* the two groups (single-target IAT; Exp2). The positive effect of holding a warm object was observed only on the relative evaluations (Exp1). Thus, whether experiencing physical warmth (vs. coldness) can improve implicit attitudes toward outgroup members remains unclear. Authors have discussed that the null result might be due to the lack of intergroup bias enhancement in the participants before the experiment, insufficient warmth of the object, or the evaluation power of the single-target IAT.

The current study aimed to investigate the effects of physical warmth on implicit attitudes toward outgroup members by including intergroup bias enhancement and controlling environmental conditions (e.g., the warmth of the cup and indoor temperature). The experiment was conducted in a Japanese–Chinese intergroup context where intergroup conflict is salient [8]. After the Japanese participants had held warm or cold cups, their implicit attitudes toward imagined Chinese people were measured using the single-target IAT [9]. Further, their helping behaviors toward a Chinese individual were investigated to confirm the outcomes of these attitudes in a natural interactive manner.

## Main text

### Methods

#### Participants

Sixty-seven Japanese undergraduate and graduate students from Kyushu University (30 males, 37 females, *M*_age_ = 20.61, *SD* = 1.26) participated in the experiment for ¥1000 each (see Additional file 1). Two additional participants were excluded from the analysis due to experimental errors.

#### Materials and procedure

The experiment was conducted by two male experimenters, one Japanese and the other Chinese. To ensure that the participants could easily recognize their nationalities, the experimenters wore nametags with Japanese *kanji* (for the Japanese experimenter) and Chinese simplified characters (for the Chinese experimenter) around their necks. The Japanese experimenter handled all experimental materials (e.g., questionnaires, cups) and gave instructions in Japanese. During the experiment, the Chinese experimenter remained seated in a private cubicle and only spoke during a brief self-introduction session at the beginning of the experiment and the Helping Task, and was fully blind to experimental conditions (i.e., which participant received a warm or cold cup). The ambient conditions of the experiment are described in Additional file 1.

The participants came to the laboratory alone or in groups of two to six. They sat in separated cubicles, each containing a computer on a desk, and received the instruction that they were required to complete two independent tasks: an “object-evaluation task” and a “momentary judgment task.” The following tasks were conducted systematically.

*Intergroup bias enhancement* The participants were asked to write a recent self-relevant negative event, a task introduced to induce intergroup bias in the participants, as intergroup bias tends to be stronger in the context of an ego threat [10].

**Fig. 1:**
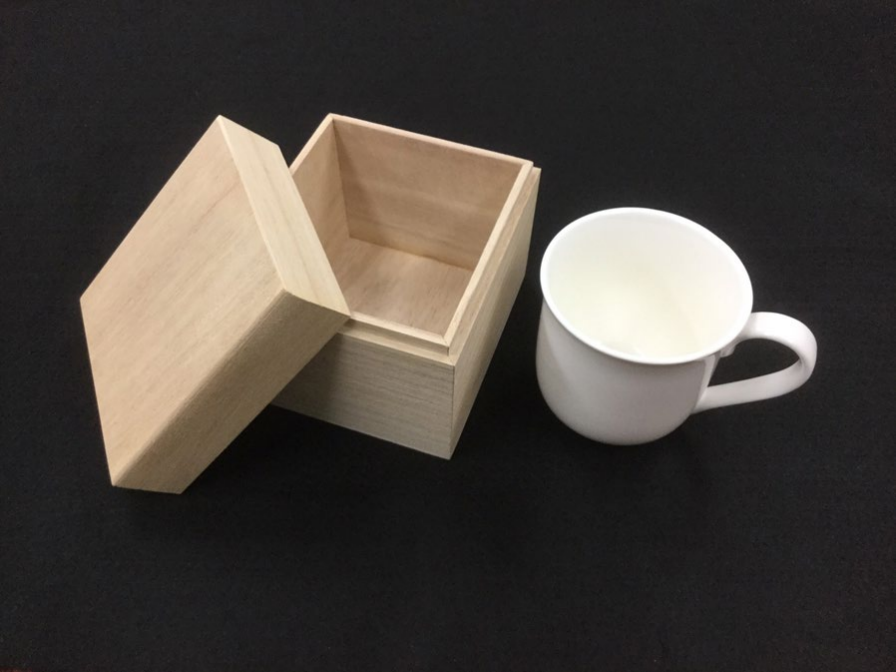
The cup and wooden box used in the experiment

*Manipulation of physical temperature (the “object*-*evaluation task”)* The participants were given an opaque wooden box with a cup inside (Fig. 1). The experimenter asked them to take the cup out and evaluate it by filling an index (a filler task; see Additional file 1), while holding the cup for 3 min. In the warm condition, the cup was filled with hot water and emptied after a few minutes. In the cold condition, the cup was placed in the freezer for 30 min. The participants were randomly assigned to either the warm or cold temperature group (*N*_warm_ = 35, *N*_cold_ = 32).

*Single*-*target IAT (the* “*momentary judgment task”)* The participants quickly classified words (i.e., positive and negative words and Chinese names) depending on their categories, which was designed to assess implicit attitudes toward Chinese people. D scores were used as an index of attitude towards Chinese (for more details, see Additional file 1).

The participants completed a Japanese version of the Positive and Negative Affect Schedule (PANAS; see Additional file 1) [11], provided their number of Chinese friends and demographic variables (e.g., gender), and rated the warmth of the cup on a 10-point scale. The experimenter announced that this was the final portion of the experiment and that he had to retrieve the receipts for their compensation from another room. He asked the participants to wait in silence until he returned and proceeded to leave the laboratory. Thus, only the Chinese experimenter and the participants were left in the laboratory.

*Helping task* The Chinese experimenter asked the participants to take part in extra tasks (3 min each) for his own study. He noted that it would be beneficial if the participants could take part in as many tasks as possible for better data collection, and emphasized that these tasks had nothing to do with the compensation of the current experiment. He then asked the participants to write down the number of tasks in which they could take part and whether they had time in their schedule after the experiment. This procedure helped identify whether low help was due to low willingness to cooperate or merely because of conflicting schedules. No further information regarding the extra tasks was provided. After the helping task, the Japanese experimenter returned to the laboratory and thoroughly debriefed, paid, and thanked the participants. No extra experiments were actually conducted with the participants.

For the statistical analyses, a t test (or analysis of covariance (ANCOVA) for comparisons of more than two groups or on multi-levels) and an Exact Wilcoxon rank sum test were conducted for the group comparisons of data that had and did not have a normal distribution, respectively. All reported p values are two-tailed. A p < .05 was considered significant.

### Results

#### Manipulation of physical temperature

Participants in the warm condition (*M* = 9.23, *SD* = .90) rated the cup significantly warmer than those in the cold condition (*M* = 1.38, *SD* = .60; *W* = 1120, *p* < .001; Exact Wilcoxon rank sum test). This indicated that the manipulation of physical temperature was successful.

#### IAT score

We conducted a 2 (condition: warm vs. cold) × 2 (gender: male vs. female) between-subject ANCOVA, including the number of Chinese friends in the model as a covariate. Although there was no direct evidence to predict that gender would modulate the effects of physical warmth on the implicit attitudes toward an outgroup member, since biobehavioral reaction patterns to non-friendly others could be gender-specific [12], we included the aspect of gender to the model. Moreover, because cross-group friendships are associated with more positive intergroup attitudes [13], the number of Chinese friends was controlled as a covariate.

**Fig. 2:**
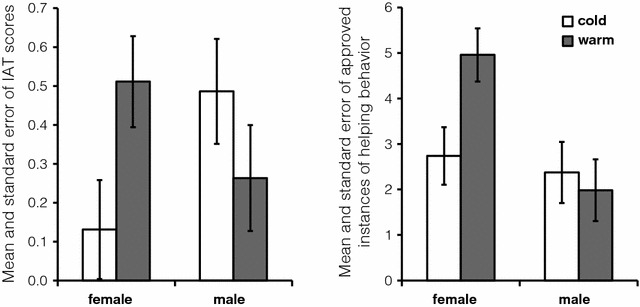
The results of IAT-scores (left) and helping behaviors (right). For both panels, error bars represent SEM

The effect of the covariate was not significant (*F*[1, 62] = .03, *p* = .87, η_p_^2^ = .00). The analysis revealed no significant main effect of the condition (*F*[1, 62] = .38, *p* = .54, η_p_^2^ = .01) or gender (*F*[1, 62] = .17, *p* = .68, η_p_^2^ = .00), but there was a significant interaction between them (*F*[1, 62] = 5.54, *p* = .02, η_p_^2^ = .08; Fig. 2). Female participants in the warm condition showed higher D scores on IAT (*M* = .51, *SD* = .45) than those in the cold condition (*M* = .13, *SD* = .52, *t*[62] = − 2.21, *p* = .03, *d* = .99). However, no difference was noted for the male participants (warm condition: *M* = .27, *SD* = .63; cold condition: *M* = .49, *SD* = .47; *t*[62] = 1.17, *p* = .25, *d* = .43).

#### Helping behavior

We performed a 2 (condition: warm vs. cold) × 2 (gender: male vs. female) ANCOVA, as described above. Additionally, since the presence of other people inhibits helping behavior [14], we also controlled the group size (i.e., the number of people that participated in the experiment together) as a covariate. The results showed that participants in a larger group offered less help (*F*[1, 61] = 7.38, *p* < .01, η_p_^2^ = .11), but no significant effect was observed in the number of Chinese friends the participants had (*F*[1, 61] = .56, *p* = .46, η_p_^2^ = .01). The analysis revealed no significant main effect of the condition (*F*[1, 61] = 2.06, *p* = .16, η_p_^2^ = .03; Fig. 2) but of gender (*F*[1, 61] = 6.43, *p* = .01, η_p_^2^ = .10), and a significant interaction of condition and gender (*F*[1, 61] = 4.19, *p* = .04, η_p_^2^ = .06). Female participants in the warm condition (*M* = 4.96, *SD* = 4.08) had a better helping behavior than those in the cold condition (*M* = 2.74, *SD* = 1.93, *t*[61] = −2.60, *p* = .01, *d* = 1.16); however, this was not the case amongst the male participants (warm condition: *M* = 1.98, *SD* = 1.85; cold condition: *M* = 2.37, *SD* = 1.60; *t*[61] = .41, *p* = .68, *d* = .15; also see Additional file 1 for additional analyses).

### Discussion

The current research provided the first piece of evidence supporting the idea that experiencing physical warmth (vs. coldness) positively impacts one’s attitude and helping behavior toward outgroup members (see related topic toward in-group members [1, 15]), although only among female participants. Japanese participants’ attitude toward Chinese people and helping behaviors toward the Chinese experimenter were significantly correlated (*r* = .34, *p* = .04, in the female participants), which supports the effects of enhanced implicit attitudes and further suggests that experiencing physical warmth could also modulate the prosocial response to outgroup members.

A previous study revealed the effects of experiencing physical warmth on attitudes toward out-group *relative to* in-group (through dual-target IAT), but not *directly toward* the two groups (through single-target IAT; [7]). As extensions to these findings, the current results seem to reduce the possibility that the single-target IAT is insufficient to evaluate modulated attitude toward outgroup members through experiencing physical warmth, while increase the possibility that intergroup bias enhancement plays an important role in promoting these effects. Consistent with this view, a previous study has demonstrated that the effects physical warmth on social affiliation depends on the situation in which the warmth is experienced; people who had been primed with a physical threat (as compared with control conditions) responded to warmth with stronger increases in affiliative motivation [16]. In this sense, however, one might ask whether the current contexts could also elicit the same effects (including the gender-depended response) in participants toward in-group members. Further investigations, including systematic combinations of gender and nationalities (i.e., group identities) of the experimenters and participants are needed for an integrative discussion to understand the mechanism of the effects of experiencing physical warmth on intergroup bias.

Despite the absence of a correlation between IAT scores and helping behavior in male participants, the main effect of gender was only observed in the latter measure. This might not be surprising, considering that females are somewhat more likely to help others [17, 18]. It might also be valuable to examine whether gender (e.g., females’ “tend and befriend” (as opposed to “fight and flight” in males) reaction to non-friendly others [12]) modulates the effect of experiencing physical warmth on evaluations toward outgroup members, which might be reflected by the reverse pattern in males to female participants in their response in the IAT scores [19].

## Limitations

Although we empirically demonstrated that experiencing physical warmth could affect interpersonal warmth, whether physical warmth increased, physical coldness reduced, or both affected the attitudes and helping behaviors toward outgroup members was unclear. It might be worth adding a control condition (e.g., neutral temperature) during further examinations.

## Additional file

**Additional file 1.** Methodological details.

## Abbreviations

IAT: implicit association test
ANCOVA: analysis of covariance
