# Supplementary material for: Experiencing physical warmth affects implicit attitudes and altruistic behavior toward outgroup in females
Source: BMC Res Notes. 2017 Nov 29;10:648. doi: 10.1186/s13104-017-2972-3 (PMC5707871; doi:10.1186/s13104-017-2972-3)
Supplement: Supplementary file 1 — Additional file 1. Methodological details. [file 13104_2017_2972_MOESM1_ESM.docx]

**Additional information**

**The sample size of the experiment**

The sample size was determined by the number of participant that could be recruited, which was sufficient considering previous research [1]. Further, the post-hoc power analysis showed acceptable values of power for the significant interactions between condition and gender in the IAT Score (0.66) and Helping Behavior (0.77). Together, the current sample size is considered sufficient to reject the null hypothesis (i.e., obtaining a significant result) when the real differences are equal to the effect size that was revealed in the current study.

**The ambient conditions of the experiment**

The room temperature and humidity were kept at approximately 27℃ (*M* = 26.72, *SD* = 0.34, ranged from 25.9 to 27.4℃) and 52% (*M* = 51.87, *SD* = 3.13, ranged from 46 to 60%), respectively. The outdoor temperature was at approximately 28℃ (*M* = 28.07, *SD* = 1.46, ranged from 23.8 to 29.9℃).

**Manipulation of physical temperature (the “object-evaluation task”)**

The experimenter asked the participants to take the cup out of the wooden box and evaluate it with ten adjective pairs (good/bad, high quality/low quality, like/dislike, first class/low grade, pleasant/unpleasant, attractive/unattractive, interesting/uninteresting, beautiful/ugly, comfortable to touch/uncomfortable to touch, cheap/expensive) on a 10-point scale after holding the cup for three minutes. This procedure was adopted from previous literature [2]. The results showed no significant difference between the physical temperature conditions for any adjective pair (all *p* > .09, range of *t* = -1.72–0.002) except the “cheap/expensive” pair (*t*[65] = -2.26, *p* = .03, *d* = .55).

**Details of the single-target IAT**

The IAT was designed to detect the strength of implicit associations between concepts based on the idea that response fluency can be promoted when closely related items share the same response key. The stimuli to be classified included positive and negative words as well as Chinese names (as shown in the following table). The authors selected the positive and negative words from a list that was referenced from Greenwald et al. [3].

Participants were asked to quickly categorize words presented in the middle of the screen (i.e., target word) into referential categories that were listed on the left and right sides of the computer screen by pressing the “F” key if the word belonged to the category on the left and the “J” key if the word belonged to the category on the right. The following is the description of the procedure, which consisted of four phases.

In the first phase, participants completed *practice blocks* that consisted of either China-negative or China-positive blocks (18 trials). Specifically, in the China-negative blocks, Chinese names and positive or negative words were presented as the *target word*, while China and negative categories were placed on the same side (the positive category was placed on the opposite side) as the referential categories. However, in the China-positive blocks, China and positive categories were placed on the same side as the referential categories were (the negative category was placed on the opposite side). That is, participants who negatively associated with Chinese were expected to accurately sort (i.e., press the correct key) for all the *target words* (i.e., Chinese name, positive words, and negative words) faster in the China-negative than in the China-positive blocks. In the second phase, participants completed the critical blocks (54 trials) as in the first phase. In the third and the fourth phases, the inversed combinations (i.e., the position of China category) were used. The order of the China-positive and China-negative blocks was counterbalanced between participants. Further, all stimuli (i.e., Chinese names and positive and negative words) were presented in random order within the blocks. Based on the response time, the D score of each participant was calculated to assess the strength of the automatic association between categories [4]. The method of calculation was based on a previous study [5] and is available from the authors of the current study upon request. Higher D scores indicate a greater positive attitude towards China.

| Traits | | Names | |
| --- | --- | --- | --- |
| Japanese Original | English Translation | Japanese Original | English Translation |
| *Positive* |  |  |  |
| 愛情 | Love | 李寧 | Ning Li |
| 健康 | Health | 王麗 | Li Wang |
| 正直 | Honest | 劉濤 | Tao Li |
| 幸運 | Lucky | 張偉 | Wei Zhang |
| 誠実 | Sincere | 楊蘭 | Lan Yang |
| *Negative* |  |  |  |
| 事故 | Accident |  |  |
| 悪臭 | Stink |  |  |
| 嫌悪 | Hatred |  |  |
| 貧乏 | Poverty |  |  |
| 苦痛 | Pain |  |  |

**The effect of physical temperature on positive and negative affect**

Touching a warm or cold cup might result in the participant feeling positive or negative; this could affect their evaluations of both the IATs and helping behaviors. To rule out this possibility, we measured the participants’ affects after touching the cups to confirm that that they were not influenced by a change in the physical temperature.

Specifically, the participants were asked to complete the Japanese version of PANAS [6] after the IATs. Cronbach’s α values for positive and negative affect were .87 and .85, respectively. The results showed that condition had no significant effect on either positive affect (*M_warm_* = 3.01, *SD_warm_* = 0.95; *M_cold_* = 3.13, *SD_cold_* = 0.86; *t*[65] = 0.55, *p* = .59, *d* = .13) or negative affect (*M_warm_* = 2.90, *SD_warm_* = 1.13; *M_cold_* = 2.68, *SD_cold_* = 0.81; *t*[65] = -0.90, *p* = .37, *d* = .22); thus, we did not include the PANAS score in further analyses.

**Analyses on helping task excluding participants with a busy schedule**

Twelve participants (*N_warm_* = 7, *N_cold_* = 5) reported that they had some business to attend to following the main experiments. Therefore, they had to back out of taking part in the extra experiments due to their schedule and not because of low willingness to cooperate. However, the number of those who could not engage in extra experiments did not differ across conditions (*χ^2^* = 0.22, *df* = 1, *p* = .64; chi-squared test), and the effect of interaction of condition and gender did not change when those participants were excluded (*F*[1, 49] = 3.93, *p* =.053, η_p_^2^ = .07). Helping behavior in the warm condition (*M* = 5.90, *SD* = 3.82) was significantly higher than that in the cold condition, in the female participants (*M* = 3.44, *SD* = 1.56, *t*[49] = -2.81, *p* < .01, *d* = 1.41). However, there were no significant differences in helping behaviors between the warm (*M* = 2.41, *SD* = 1.79) and cold conditions (*M* = 2.53, *SD* = 1.60) in the male participants (*t*[49] = 0.13, *p* = .90, *d* = .05).

**References**

1. Williams LE, Bargh JA. Experiencing physical warmth promotes interpersonal warmth. Science. 2008; doi:10.1126/science.1162548.
2. Wei W, Ma J, Wang L. The ‘warm’side of coldness: cold promotes interpersonal warmth in negative contexts. Br J Soc Psychol. 2015; doi:10.1111/bjso.12108.
3. Greenwald AG, McGhee DE, Schwartz JL. Measuring individual differences in implicit cognition: The implicit association test. J Pers Soc Psychol. 1998;74(6):1464-1480.
4. Greenwald AG, Nosek BA, Banaji MR. Understanding and Using the Implicit Association Test: I. An Improved Scoring Algorithm. J Pers Soc Psychol. 2003; doi:10.1037/0022-3514.85.2.197.
5. Karpinski A, Steinman RB. The single category implicit association test as a measure of implicit social cognition. J Pers Soc Psychol. 2006; doi:[10.1037/0022-3514.91.1.16](http://psycnet.apa.org/doi/10.1037/0022-3514.91.1.16)
6. Sato A, Yasuda A. Development of the Japanese version of Positive and Negative Affect Schedule (PANAS) scales. Jpn J Pers. 2001;9:138-139.
